# CXCR4-expressing *Mist1*^+^ progenitors in the gastric antrum contribute to gastric cancer development

**DOI:** 10.18632/oncotarget.22451

**Published:** 2017-11-10

**Authors:** Kosuke Sakitani, Yoku Hayakawa, Huan Deng, Hiroshi Ariyama, Hiroto Kinoshita, Mitsuru Konishi, Satoshi Ono, Nobumi Suzuki, Sozaburo Ihara, Zhengchuan Niu, Woosook Kim, Takayuki Tanaka, Haibo Liu, Xiaowei Chen, Yagnesh Tailor, James G. Fox, Stephen F. Konieczny, Hiroshi Onodera, Antonia R. Sepulveda, Samuel Asfaha, Yoshihiro Hirata, Daniel L. Worthley, Kazuhiko Koike, Timothy C. Wang

**Affiliations:** ^1^ Division of Digestive and Liver Disease, Department of Medicine, Columbia University, College of Physicians and Surgeons, New York, NY, USA; ^2^ Graduate School of Medicine, The University of Tokyo, Department of Gastroenterology, Tokyo, Japan; ^3^ Department of Pathology, The Fourth Affiliated Hospital of Nanchang University, Nanchang, China; ^4^ Institute for Adult Diseases, Asahi Life Foundation, Tokyo, Japan; ^5^ Department of General Surgery, Zhongshan Hospital, Fudan University, Shanghai, China; ^6^ Division of Comparative Medicine, Massachusetts Institute of Technology, Boston, MA, USA; ^7^ Department of Biological Sciences, The Purdue Center for Cancer Research, Purdue University, West Lafayette, IN, USA; ^8^ Department of Electrical and Electronic Engineering, The University of Tokyo, Tokyo, Japan; ^9^ Division of Clinical Pathology and Cell Biology, Department of Pathology, Columbia University College of Physicians and Surgeons, New York, NY, USA; ^10^ Cancer theme, SAHMRI and Department of Medicine, University of Adelaide, SA, Australia

**Keywords:** gastric cancer, stem cell, mist1, cxcr4, cxcl12

## Abstract

*Mist1* was recently shown to identify a discrete population of stem cells within the isthmus of the oxyntic gland within the gastric corpus. Chief cells at the base of the gastric corpus also express *Mist1*. The relevance of *Mist1* expression as a marker of specific cell populations within the antral glands of the distal stomach, however, is unknown. Using *Mist1*-CreERT mice, we revealed that *Mist1*^+^ antral cells, distinct from the *Mist1*^+^ population in the corpus, comprise long-lived progenitors that reside within the antral isthmus above *Lgr5*^+^ or CCK2R^+^ cells. *Mist1*^+^ antral progenitors can serve as an origin of antral tumors induced by loss of Apc or MNU treatment. *Mist1*^+^ antral progenitors, as well as other antral stem/progenitor population, express Cxcr4, and are located in close proximity to Cxcl12 (the Cxcr4 ligand)-expressing endothelium. During antral carcinogenesis, there is an expansion of Cxcr4^+^ epithelial cells as well as the Cxcl12^+^ perivascular niche. Deletion of Cxcl12 in endothelial cells or pharmacological blockade of Cxcr4 inhibits antral tumor growth. Cxcl12/Cxcr4 signaling may be a potential therapeutic target.

## INTRODUCTION

Gastric cancer is one of the leading causes of cancer death worldwide, and the prognosis for patients with advanced disease remains poor [1, 2]. Gastric cancer contains multiple histological subtypes with distinct molecular signatures, but dominant oncogenic mutations are generally less frequent in gastric cancers than in other gastrointestinal cancers [3], limiting opportunities for targeted therapy in the disease. Cancer growth is influenced greatly by interactions between cancer stem cells and the tumor microenvironment, which has emerged as a promising therapeutic target [4, 5]. Cancer stem cells are believed to arise from normal stem or progenitor cells [6]. Stem cells are defined by the properties of self-renewal and multi-potency, or the ability to give rise to more than one lineage, which are modulated to some extent by the surrounding microenvironment or niche [7, 8].

The mouse stomach is comprised of three major parts, forestomach, corpus, and antrum. Forestomach consists of squamous epithelium and thus is in many aspects more similar to esophageal epithelium. The proximal part corpus and distal part antrum have columnar epithelium and are collectively referred to the glandular stomach. We recently showed that relatively quiescent *Mist1*^+^ gastric corpus stem cells located in the isthmus, where the corpus gland narrows near the upper third position of glands, can serve as the cellular origin of all epithelial lineages, as well as gastric cancer. In the corpus, *Mist1*^+^ stem cells are regulated by Cxcl12^+^ endothelial cells and Cxcr4^+^ innate lymphoid cells (ILCs), and the Cxcl12/Cxcr4 niche is required for progression to diffuse-type gastric cancer [9]. During inflammation, the Cxcl12/Cxcr4 niche expands and supports cancer cell growth by paracrine release of growth factors such as Wnt5a. However, the gastric corpus and antrum are two different organs, in terms of not only anatomical and functional differences but also their stem cell biology and contribution to carcinogenesis. Moreover, intestinal-type gastric cancer is typically more common in *Helicobacter pylori*-related human gastric cancers than diffuse-type. Therefore, the contribution of Cxcl12/Cxcr4 in antral stem cells and other form of gastric cancer has not been fully elucidated.

In the gastric antrum, several stem/progenitor cell markers have been identified such as CCK2R, Sox2, eR1, *Villin, Axin2,* and *Lgr5* [10–15]. *Lgr5*^+^ cells are present at the base of antral glands, whereas other stem/protenitor cells reside within the antral isthmus, where gland narrows just above *Lgr5*^+^ cells. Although Wnt and Notch signaling have been suggested as important modulators of gastrointestinal stem cells, other critical niche signals that regulate antral stem cells, and that might contribute to the development of cancer, have not been fully explored. Here, we identify *Mist1* expression in antral isthmus progenitors, and define their contribution to the antral lineages and to gastric cancer. Finally, we demonstrate a role for Cxcl12/Cxcr4 signaling in antral tumorigenesis.

## RESULTS

### *Mist1* is expressed in long-lived isthmus progenitors in the antrum

**Figure 1:**
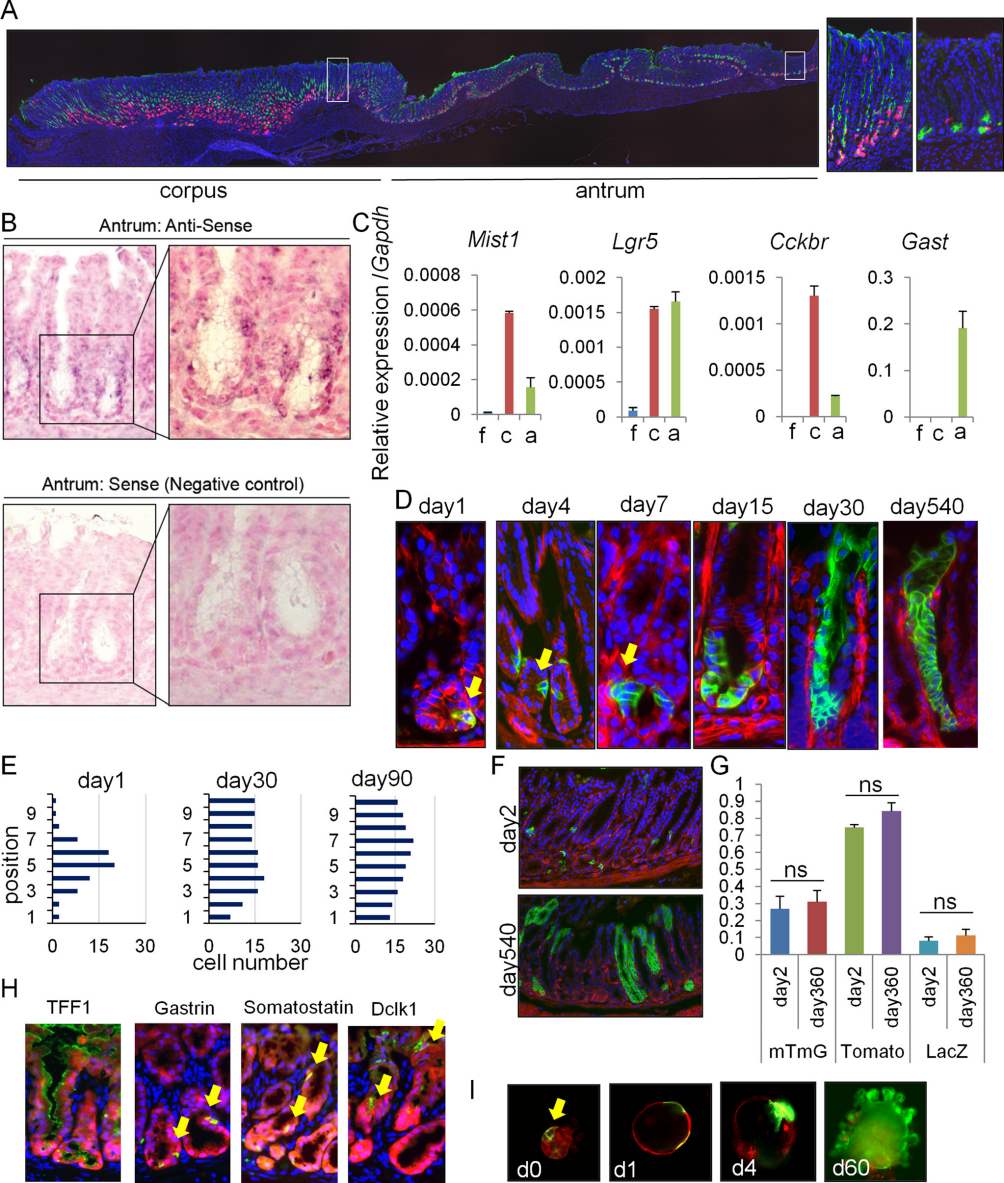
*Mist1* marks long-lived, multipotent isthmus progenitors in the antrum (**A**) Longitudinal stomach section of *Mist1*-CreERT; *R26*-TdTomato mice stained with GS-II (green). Areas indicated by white boxes in the corpus and antrum are enlarged in right. Macroscopic cut line of the section is shown by blue line in S1D. (**B**) In situ hybridization of *Mist1* in the antrum. (**C**) Relative gene expression per *Gapdh* in each part of the stomach (*n* = 3). (**D**–**F**) Lineage tracing in *Mist1*-CreERT; *R26*-mTmG mice from days 1-540. Arrows indicate *Mist1*^+^ cells and their progeny. Quantification of the *Mist1*-traced cell position is shown in (E). A total of 50 glands were analyzed at each time point. (**G**) Lineage tracing frequency in *Mist1-*CreERT; *R26*-mTmG, *Mist1*-CreERT; *R26*-TdTomato, and *Mist1*-CreERT; *R26*-LacZ mice at day 1 and 360. (**H**) Immunofluorescence of the indicated markers (green) in *Mist1*-CreERT; *R26*-TdTomato mice 12 months after TAM induction. (**I**) Antral gland culture of TAM-induced *Mist1*-CreERT; *R26*-mTmG mice. The arrow indicates *Mist1*^+^ cells.

Although *Mist1* expression has been reported in the gastric corpus [16], we recently found abundant Cre recombination in the antrum of *Mist1*-CreERT mice, and that *Mist1* antral lineages appeared to contribute to tumor development [17]. To clarify *Mist1* expression in the stomach in more detail, we looked at an entire longitudinal section of *Mist1*-CreERT; *R26*-TdTomato mice 5 days after tamoxifen (TAM) induction (Figure 1A). As reported previously [9], there are abundant TdTomato^+^ chief cells at the corpus gland base, while there are TdTomato^+^ stem cells above GSII^+^ mucous neck region, some of which start to lineage trace from the corpus isthmus. In addition, we observed scattered, but solid TdTomato expression within the antral isthmus where stem/progenitors are thought to reside, in contrast to previous reports [16]. These cells reside adjacent to GSII-expressing deep antral mucous cells, but most of them do not overlap. In addition, these TdTomato^+^ cells are negative for other differentiated cell markers found in this region, including Dclk1, somatostatin, and gastrin (Supplementary Figure 1A). In situ hybridization confirmed *Mist1* mRNA expression in cells at this position (Supplementary Figure 1B–1C), while Mist1 protein was not detected by immunohistochemical staining (not shown). We performed RT-PCR using mRNA extracted from different parts of the stomach including forestomach, corpus, and antrum, and confirmed that *Mist1* is expressed in the antrum, at a lower level compared to the corpus, but a higher level compared to the forestomach, where no TdTomato^+^ cells are seen (Supplementary Figure 1C, 1D). A gastrin receptor gene *Cckbr*, which is expressed in differentiated cells in the corpus and stem cells in the antrum, is also expressed in these parts similar to *Mist1*. *Lgr5* expression is equivalent between corpus and antrum, as reported previously [18]. Thus, *Mist1* expression level well correlates with recombination rate in each part of the stomach of *Mist1*-CreERT mice.

We next performed a detailed time course of lineage tracing in *Mist1*-CreERT; *R26*-mTmG mice. The *Mist1*-CreERT; *R26*-mTmG mice showed isolated recombined GFP^+^ cells in the lower third of antral glands at 1 day after TAM induction (Figure 1D–1E). *Mist1*^+^ cells were present at average of 1-2 cells/gland, ranging from position 1 to 7 with a peak at position +5. After TAM induction, the *Mist1*^+^ lineage expanded gradually with a doubling time of ∼4 days, and entire antral glands were labeled within 30 days (Figure 1D–1E). Similar lineage tracing pattern in antral glands was also observed in *Mist1*-CreERT; *R26*-LacZ and *Mist1*-CreERT; *R26*-TdTomato mice (Supplemantary Figure 1E–1F). Lineage tracing in these mice persisted beyond 18 months, and the *Mist1* lineage contained all cell types including TFF1^+^ surface pit cells, gastrin^+^ G cells, somatostatin^+^ D cells, and Dclk1^+^ tuft cells. (Figure 1H). The frequency of lineage tracing was consistent throughout the observation period, depending on the reporter strain used (Figure 1G). We did not observe any tamoxifen-induced epithelial injury in the antrum during the time course, as reported previously [19]. Lineage tracing during *in vitro* organoid culture supported an expansion of the *Mist1*^+^ lineage (Figure 1I). Together, these results suggest that *Mist1*-expressing antral cells contain long-lived, multipotent progenitors in the isthmus.

### *Mist1*^+^ cells take up BrdU more rapidly than *Lgr5*^+^ cells in the antrum

**Figure 2:**
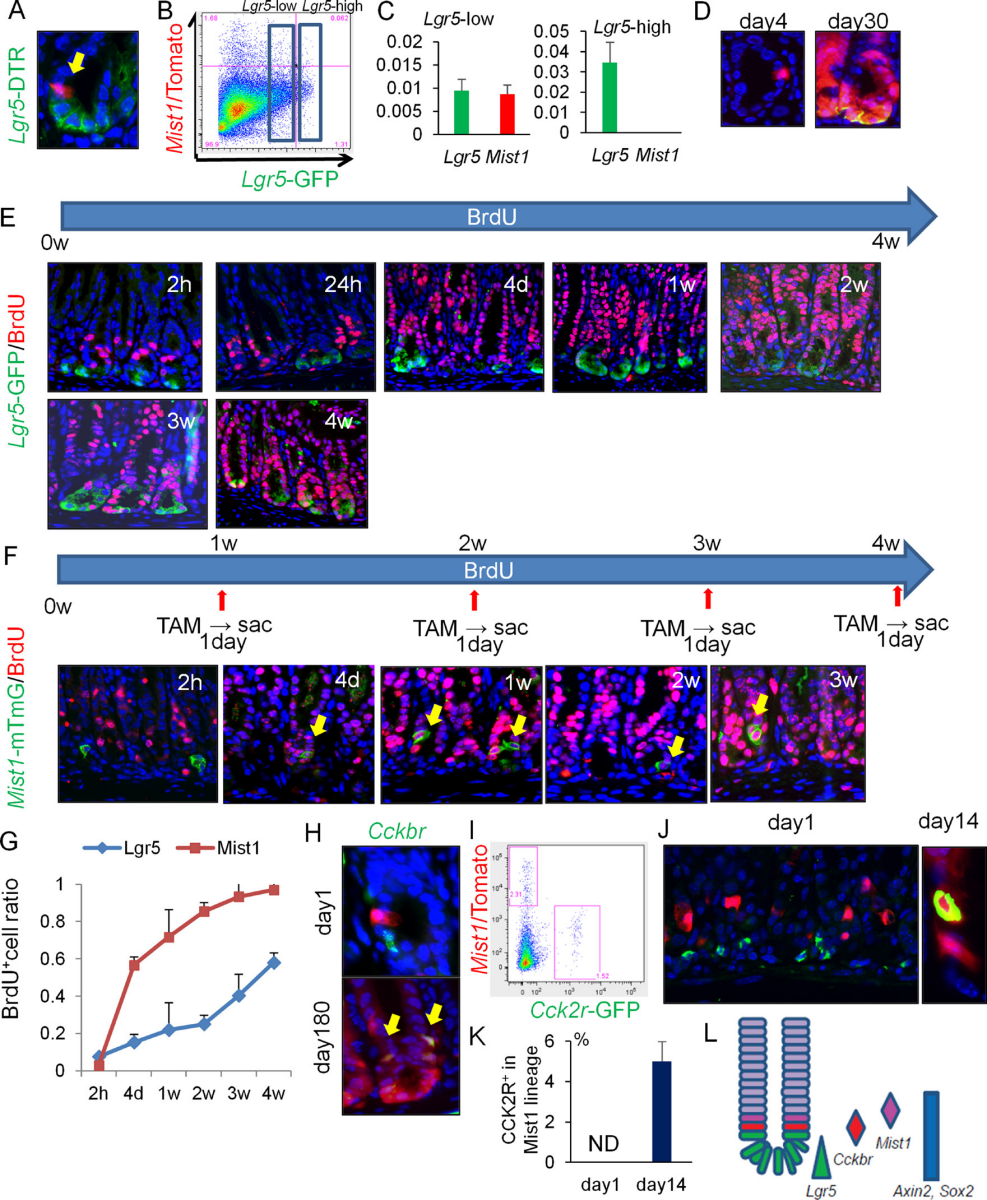
*Mist1*^+^ cells take up BrdU more rapidly than *Lgr5*^+^ cells (**A**) *Lgr5* (green) and *Mist1* (red) expression in *Mist1*-CreERT; *Lgr5*-DTR-GFP; *R26*-TdTomato mice 1 day after TAM induction. (**B**) FACS plot of *Mist1*-CreERT; *Lgr5*-DTR-GFP; *R26*-TdTomato mice antrum 1 day after TAM induction. Boxes indicate *Lgr5*-low and *Lgr5*-high expressing cell populations. (**C**) Relative mRNA expression/*Gapdh* of *Lgr5* and *Mist1* in *Lgr5*-high cells and *Lgr5*-low cells from the *Mist1*-CreERT; *Lgr5*-DTR-GFP; *R26*-TdTomato mice antrum 1 day after TAM induction. N.D. means “not detected”. *N* = 3. (**D**) Lineage tracing of DT-treated (day4 and day30 after tamoxifen induction) *Mist1*-CreERT; *Lgr5*-DTR-GFP; *R26*-TdTomato mice. DT was given at 1 day after tamoxifen. (**E**) Immunofluorescence of GFP (green) and BrdU (red) in *Lgr5*-EGFP-IRES-CreERT mice given BrdU continuously by drinking water (1.0 mg/ml). Mice were sacrificed at indicated time points. (**F**) Immunofluorescence of GFP (green) and BrdU (red) in *Mist1*-CreERT; *R26*-mTmG mice given BrdU continuously by drinking water. Mice were sacrificed at the indicated time points (1 day after TAM induction). (**G**) BrdU^+^ cell ratio of *Lgr5*^+^ cells and *Mist1*^+^ cells. A total of 300 cells from three mice were analyzed at each time point. (**H**) Antral images of *Mist1*-CreERT; *R26*-TdTomato mice crossed to *Cckbr*-EGFP mice 1 and 180 days after TAM induction. Arrows indicate Tomato and EGFP double-positive cells. (**I**) FACS plot of *Mist1*-CreERT; *R26*-TdTomato; *Cckbr*-EGFP mice 1 day after tamoxifen. (**J**–**K**) Immunofluorescence images showing CCK2R staining (green) in *Mist1*-CreERT; *R26*-TdTomato mice 1 day and 14 days after TAM induction. CCK2R^+^ cells/*Mist1*^+^ cells are quantified in (K). A total of 300 cells from three mice were analyzed. (**L**) Schematic model of antral stem/progenitor cells.

Given that *Lgr5* expression has also been associated in the antrum with long-lived, self-renewing stem cells [10], we examined possible overlap between *Mist1* and *Lgr5* using *Mist1*-CreERT; *Lgr5*-DTR-GFP; *R26*-TdTomato mice. Although *Mist1*^+^ cells and *Lgr5*^+^ cells are often located in close proximity, *Mist1*^+^ cells were again located slightly higher up in the antral glands, and the vast majority (e.g. > 95%) of *Mist1*^+^ cells were found to be *Lgr5*-negative by microscopic and FACS analysis (Figure 2A–2B). We sorted *Lgr5*-high and *Lgr5*-low expressing cells separately, and confirmed that *Mist1* mRNA is expressed in *Lgr5*-low cells, but not in *Lgr5*-high cells (Figure 2C). We then ablated *Lgr5*^+^ cells by administration of diphtheria toxin (DT), and found that lineage tracing of *Mist1*^+^ cells still occurred with unchanged kinetics (Figure 2D). Furthermore, the *Lgr5*^+^ cells reappeared by 30 days within the *Mist1*^+^ lineage, indicating that *Mist1*^+^ stem cells can give rise to *Lgr5*^+^ cells. Taken together, *Mist1*^+^ antral stem cells are mostly distinct from *Lgr5*^+^ cells.

To further analyze the differences between *Mist1*^+^ and *Lgr5*^+^ antral cells, we administered bromodeoxyuridine (BrdU) to *Lgr5-*EGFP-IRES-CreERT mice and *Mist1-*CreERT; R26-mTmG mice continuously through their drinking water. *Lgr5*-high cells at the base of glands (positions +1 to +3) failed to label with BrdU in the first few weeks, while *Lgr5*-low cells in the isthmus occasionally took up BrdU and then such labeling expanded bi-directionally (Figure 2E). However, it required more than 4 weeks to label all basal *Lgr5*^+^ cells with continuous BrdU administration, suggesting that many of these cells are either quiescent or post-mitotic. Indeed, using a FucciG1 transgenic mouse line where cells in G1 cell cycle state turns into red and cells in post-mitotic cells show bright red signal due to accumulation of fluorescent protein after cell cycle exit [20], we realized that most of *Lgr5*-high cells in the antrum of *Lgr5*-EGFP-IRES-CreERT; FucciG1 mice display bright red nuclei thus they are likely post-mitotic (Supplementary Figure 2A).

In contrast, while *Mist1*^+^ cells did not take up BrdU immediately, consistent with their doubling time of 4 days, they could be uniformly labeled with BrdU within two weeks (Figure 2F, 2G). Thus, these data suggest marked differences in cellular kinetics between *Mist1*^+^ and *Lgr5*^+^ cells, with the *Mist1*^+^ cells labeling with BrdU well before *Lgr5*-high cells.

To investigate possible overlap between *Mist1*^+^ cells and Sox2^+^ or CCK2R^+^ antral stem cells, we generated *Mist1*-CreERT; *Sox2*-EGFP; *R26*-TdTomato mice and *Mist1*-CreERT; *Cckbr*-EGFP; *R26*-TdTomato mice [11, 12]. One day after TAM induction, *Mist1*^+^ cells were distinct from *Cckbr*-GFP^+^ and *Sox2*-GFP^+^ cells based on immunofluorescent and FACS analysis (Figure 2H–2I, Supplementary Figure 2B–2C). Immunohistochemistry confirmed that CCK2R^+^ cells were located at the +4 position, just below *Mist1*^+^ cells (Figure 2J–2K). Axin2 protein, a recently reported antral stem cell marker, is expressed in the isthmus, but there is no overlap with *Mist1*^+^ cells (Supplementary Figure 2D). *Mist1*^+^ cells were able to generate Sox2^+^ and CCK2R^+^ cells and indeed *all* epithelial lineages within the antral gland, suggesting *Mist1* also labels *bona fide* antral stem cells, although interconversion between these various states cannot be excluded (Figure 2H, 2J–2L, Supplementary Figure 2C).

### Antral *Mist1*^+^ cells serve as a cellular origin of cancer

**Figure 3:**
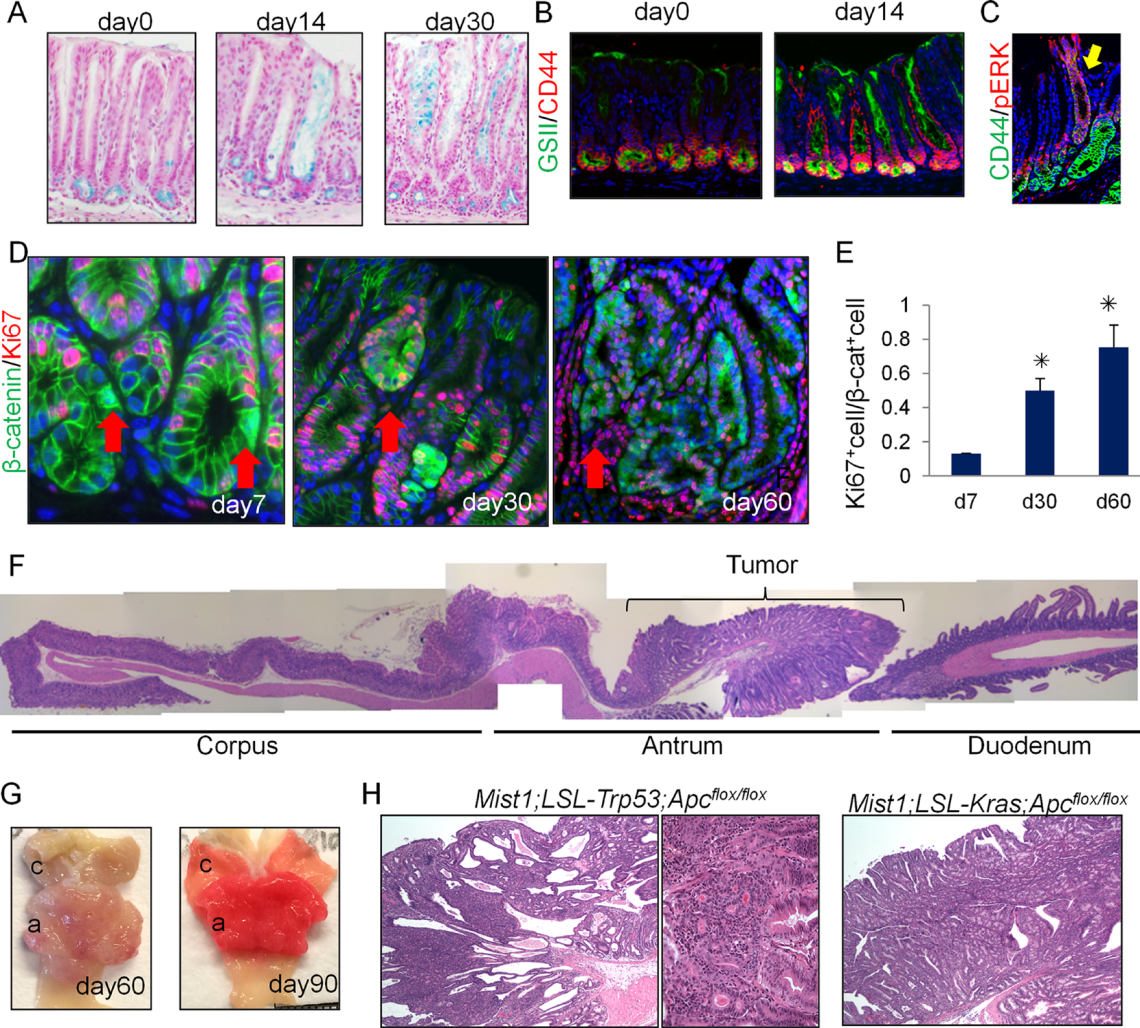
Antral *Mist1*^+^ cells serve as a cellular origin of cancer (**A**) Alcian blue (blue) staining in *Mist1*-CreERT; LSL*-Kras*^G12D^ mice at the indicated time points. (**B**) GSII (green) and CD44 (red) staining in *Mist1*-CreERT; LSL*-Kras*^G12D^ mice at the indicated time points. (**C**) CD44 (green) and p-ERK (red) staining in *Mist1*-CreERT; LSL*-Kras*^G12D^ mice at day 30 after tamoxifen. (**D**-**E**) Immunofluorescence for β-catenin (green) and Ki67 (red) in *Mist1*-CreERT; *Apc*^flox/flox^ mice on days 7, 30, and 60 after TAM induction (D). The arrows indicate the nuclear β-catenin^+^ cells. Ki67^+^ cell ratio in total nuclear β-catenin^+^ cells is quantified (E). A total of 300 cells from three mice are analyzed at each time point. (**F**) Longitudinal H&E stained section of *Mist1*-CreERT; *Apc*^flox/flox^ mouse stomach 60 days after TAM induction. (**G**) Gross picture *Mist1*-CreERT; *Apc*^flox/flox^ mice 60 and 90 days after TAM induction. (**H**) H&E staining of *Mist1*-CreERT; LSL-*Trp53*^R172H^; *Apc*^flox/flox^ mice and *Mist1*-CreERT; LSL*-Kras*^G12D^; *Apc*^flox/flox^ mice 150 days after TAM induction.

Given that *Mist1*^+^ antral cells function as stem cells in the normal stomach, we investigated whether *Mist1*^+^ stem cells could be a cell-of-origin for antral gastric metaplasia and cancer. We generated *Mist1*-CreERT; LSL-*Kras*^G12D^ mice, which were previously shown to rapidly develop gastric metaplasia of the corpus [9]. At day 14 after induction of mutant *Kras* in *Mist1*^+^ cells, we found rapid expansion from the antral gland base of mucous-producing cells that were Alcian Blue^+^, and which populated entire glands in a gland-by-gland fashion (Figure 3A). The expansion of *Kras*-activated, p-ERK^+^ cells was accompanied by an increase in CD44 and GSII expression, a marker of gastric preneoplasia [13] (Figure 3B–3C). Thus, *Kras* activation in *Mist1*^+^ antral stem cells causes antral hyperplasia with expansion of preneoplastic cells.

We next treated the *Mist1*-CreERT; *R26*-mTmG mice with the chemical carcinogen, N-nitroso-N-methylurea (MNU). One week after the 5 weekly cycles of MNU, the ratio of Ki67^+^ cells/*Mist1*^+^ cells was increased in the stomachs of MNU-treated mice compared to untreated controls, suggesting that MNU treatment activates relatively quiescent *Mist1*^+^ cells (4% are Ki67^+^ at baseline) to become more proliferative (28% are Ki67^+^ after MNU) cells (Supplementary Figure 3A). In addition, at 40 weeks MNU-derived tumors showed robust lineage tracing from *Mist1*^+^ cells (Supplementary Figure 3B) in 50 % of mice analyzed (3/6), showing that MNU-induced antral tumor is at least in part derived from *Mist1*^+^ cells.

We then crossed *Mist1*-CreERT mice with *Apc*^flox/flox^ mice and generated *Mist1*-CreERT; *Apc*^flox/flox^ mice in order to examine the effect of *Apc* loss in *Mist1*^+^ cells. As shown in Supplementary Figure 3C, 2 days after TAM induction, there was strong nuclear translocation of β-catenin evident in cells within the antral isthmus. The number of cells positive for nuclear β-catenin increased gradually, with small dysplastic nodules positive for β-catenin widely present at 14 - 30 days after the TAM induction. *Apc*-deleted *Mist1*^+^ lineages were initially Ki67-negative (day 7, 14), but later became Ki67^+^ after the formation of large dysplasia (day 30 or after) (Figure 3D–3E). Sixty days after TAM induction, ten of 10 *Mist1*-CreERT; *Apc*^flox/flox^ mice (100 %) exhibited large antral tumors, as shown previously in different Cre driver mice [21] (Figure 3E, 3F). Notably, macroscopic tumors in these mice are confined to the antrum (the incidence of corpus tumor is 0 % (10/10)), while the normal corpus is sometimes displaced proximally towards the forestomach when the tumors enlarge at later stage. Although *Mist1* is also expressed in corpus stem cells and Brunner glands in the duodenum, these tissues were unaffected by the loss of *Apc* in *Mist1*^+^ cells. Thus, in the upper GI tract, antral *Mist1*^+^ cells appeared uniquely susceptible to *Apc*/β-catenin-driven tumorigenesis.

Histologically, high-grade, intraepithelial neoplasia/carcinoma *in situ* were seen in the antrum of *Mist1*-CreERT; *Apc*^flox/flox^ mice around 60 days after tamoxifen induction, which expanded further by day 120 but remained intra-mucosal without invasion (Supplementary Figure 3D). Antral organoids of *Mist1*-CreERT; *Apc*^flox/flox^ mice can grow without Wnt3a and R-spondin 1 in culture media only when recombination is induced by tamoxifen, suggesting that the tumor development in these mice is cell-autonomous effect (Supplementary Figure 3E, 3F). The combination of *Apc* loss and *Trp53* mutation led to a higher dysplastic grade, compared to *Apc* loss alone, but still remained intra-mucosal without invasion (Figure 3H). The addition of *Kras*^G12D^ mutation to *Apc* loss led to severe metaplasia and expansion of the neoplastic process both in the antrum to the corpus, but again submucosal invasion was not observed. The combination of *Apc* loss, *Kras* mutation, and *Trp53* mutation in *Mist1* lineage also generated dysplastic lesion resembling the tumors with *Apc* knockout and *Kras* mutation both in the antrum and corpus in 1 month, but all the mice died within a month due to pancreatic tumor formation (not shown). Overall, these findings indicate that *Mist1*^+^ cells can serve as an origin of antral intestinal-type cancer.

### Cxcl12/Cxcr4 axis contributes to antral stem cell niche

**Figure 4:**
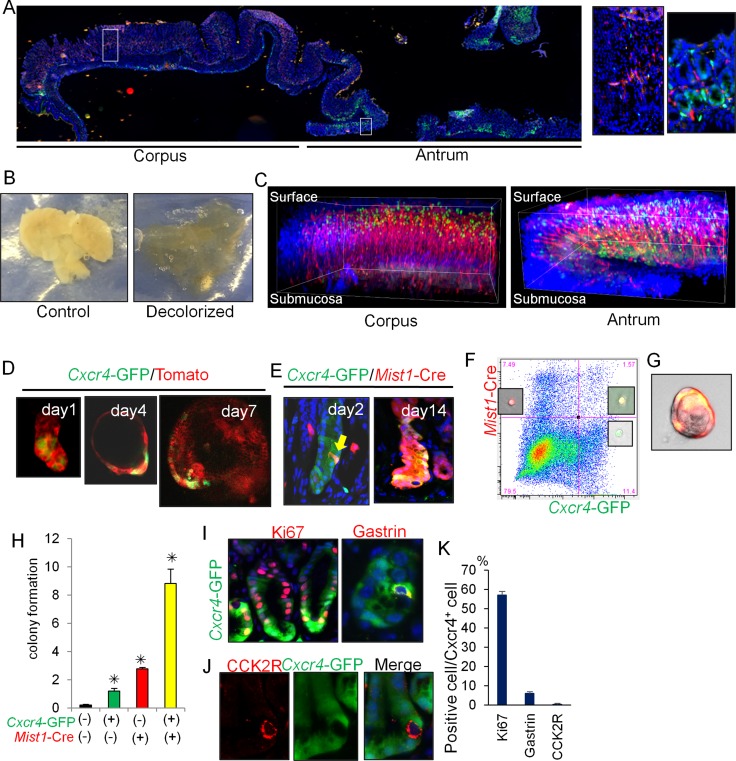
Cxcl12/Cxcr4 axis contributes to antral stem cell niche (**A**) Longitudinal stomach section of *Cxcr4*-EGFP; *Cxcl12*-dsRED mice stained with DAPI. Areas indicated by white boxes in the corpus and antrum are enlarged in right. (**B**) Representative gross images of control and decolorized stomachs. (**C**) 3D reconstructed images of *Cxcr4*-EGFP; *Cxcl12*-dsRED mouse corpus and antrum stained with DAPI. (**D**) Antral gland culture of *Cxcr4*-EGFP; *R26*-mTmG mice. (**E**) Lineage tracing of *Mist1*-CreERT; *R26*-TdTomato; *Cxcr4*-EGFP mice 2 and 14 days after TAM induction. Arrow indicates double positive cells. (**F**) FACS plot of antral cells from *Mist1*-CreERT; *Cxcr4*-EGFP; *R26*-TdTomato mice 1 day after TAM induction. Representative images of each cellular population are shown. (G-H) Single cell culture images (**G**) and relative colony formation efficacy of sorted cells (**H**). Double-negative cells, Cxcr4^+^ cells (green), *Mist1*^+^ cells (red), and double-positive cells (yellow) were analyzed. (**I**–**K**) Immunofluorescence staining of Ki67 and gastrin (I, red), and CCK2R (J, red) in *Cxcr4*-EGFP mice. Ki67^+^, Gastrin^+^, and CCK2R^+^ cell ratio in Cxcr4^+^ cells are quantified in (K). A total of 300 cells from three mice were analyzed.

We previously showed that the Cxcl12^+^ endothelium and Cxcr4^+^ ILCs regulated gastric corpus stem cells and also contributed strongly to diffuse-type gastric cancer development [9]. When we observed Cxcl12/Cxcr4 expression in the entire stomach of *Cxcl12*-dsRED; *Cxcr4*-GFP mice, Cxcr4^+^ epithelial cells were abundant at the lower half of the antral glands (positions 1-10), while Cxcr4^+^ epithelial cells were absent and instead rare Cxcr4^+^ immune cells is present in the corpus as previously reported [9, 22] (Supplementary Figure 4A). In order to visualize distinct expression pattern between these two parts more clearly, we utilized tissue decolorization method and performed 3D reconstitution of full mucosal layers (Figure 4B, 4C) [23]. Although examination of a 5-μm section does not immediately provide clear information of cell localization, 3D images demonstrate that in the corpus, Cxcr4^+^ immune cells and Cxcl12^+^ expression in the endothelium are predominantly located in the isthmus region, and that in the antrum, there are 2 distinct Cxcr4^+^ cells including basal epithelial cells and immune cells throughout the mucosa, both of which are surrounded by Cxcl12^+^ endothelial network. Immunohistochemical staining confirmed that Cxcl12^+^ cells were CD31^+^ endothelial cells, and not podoplanin^+^ lymphatic cells nor α-SMA^+^ myofibroblasts (Supplementary Figure 4B).

Cxcr4^+^ antral epithelial cells can exist in cultured organoids, with a gradual expansion during the time course (Figure 4D). We utilized *Mist1*-CreERT; *R26*-TdTomato; *Cxcr4*-EGFP mice to investigate possible overlap between Cxcr4^+^ and *Mist1*^+^ epithelial cells. Two days after TAM induction, the Tomato signal (*Mist1*^+^ cell) and the GFP signal (Cxcr4^+^ cells) were found to overlap in isolated cells near the +5 position (Figure 4E). On day 14 after TAM induction, most of the Cxcr4^+^ cells were clearly derived from the *Mist1*^+^ progeny. FACS analysis of antral cells from *Mist1*-CreERT; *R26*-TdTomato; *Cxcr4*-EGFP mice confirmed the presence of double-positive (*Mist1*^+^Cxcr4^+^) cells (Figure 4F), while there was no double-positive cells in the corpus [9]. Single cell culture analysis revealed that *Mist1*^+^Cxcr4^+^ double-positive cells showed a higher rate of colony formation compared to single-positive or double-negative populations (Figure 4G–4H).

Approximately 50% of *Cxcr4*-EGFP^+^ cells expressed Ki67 in the lower half of glands, and thus presumably included many of the active stem/progenitor cells in this region such as *Lgr5*^+^ cells (Figure 4I). Interestingly, CCK2R^+^ stem cells in the antrum were negative for *Cxcr4*-EGFP (Figure 4J–4K). Some of the gastrin-expressing (G) cells also expressed *Cxcr4-*EGFP, whereas there was no overlap at baseline between *Cxcr4*-EGFP and Dclk1^+^ tuft cells or somatostatin^+^ D-cells (not shown). We found that Cxcr4^+^CD45^+^ immune cells present in the antrum were primarily Lin^-^CD90.2^+^Cxcr4^+^ ILCs (Supplementary Figure 4C-4D). Thus, these data indicate that that *Mist1*^+^ antral progenitors are localized within the Cxcl12/Cxcr4 perivascular niche.

### Cxcl12/Cxcr4 axis contributes to antral tumor growth

**Figure 5:**
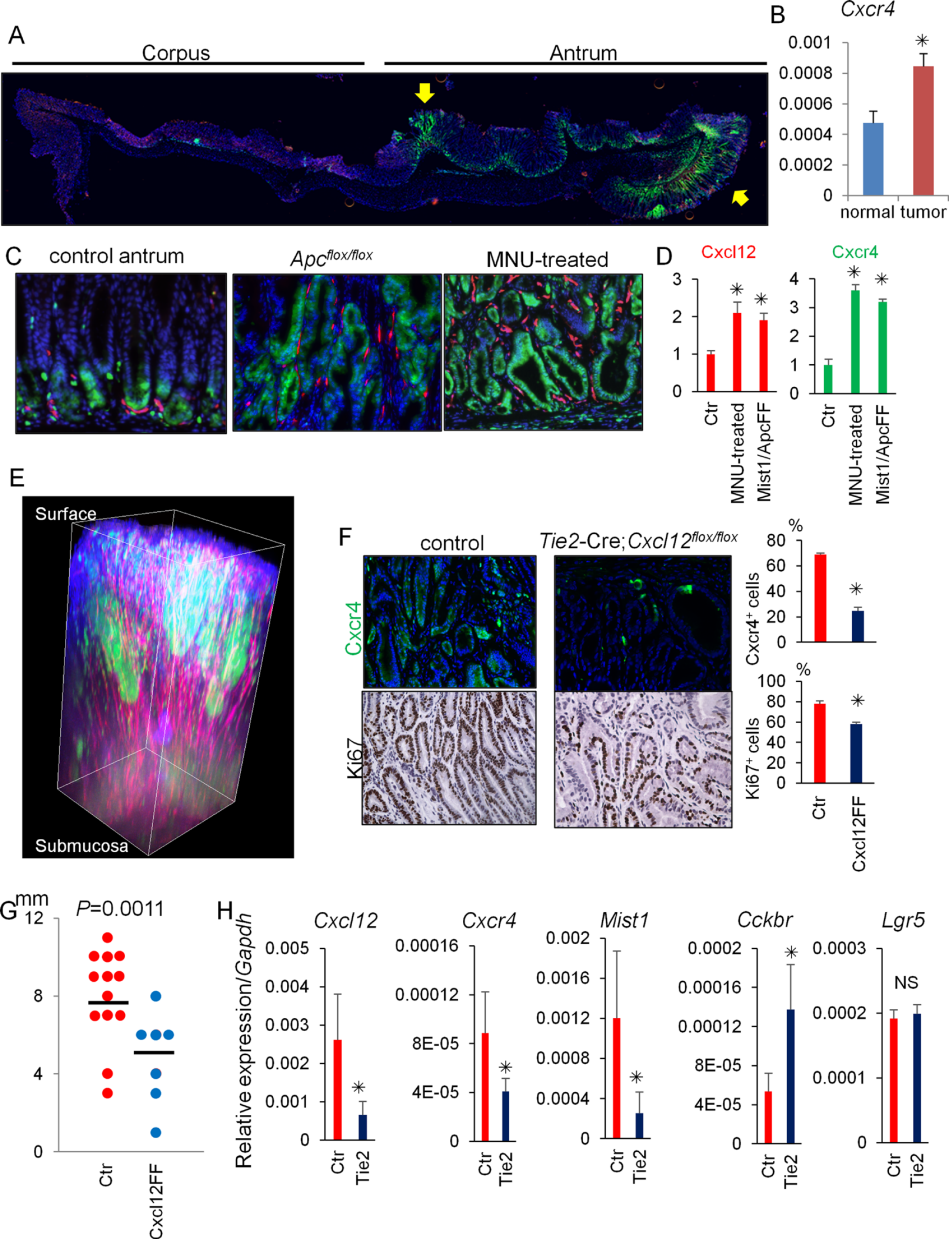
Cxcl12/Cxcr4 axis contributes to antral tumor growth (**A**) Longitudinal stomach section of *Mist1*-CreERT; *Apc*^flox/flox^; *Cxcr4*-EGFP; *Cxcl12*-dsRED mice 60 days after tamoxifen stained with DAPI. Arrows indicate dysplastic lesions. (**B**) Relative gene expression per *Gapdh* in normal antrum and antral tumors of *Mist1*-CreERT; *Apc*^flox/flox^ mice 60 days after tamoxifen. (**C**–**D**) *Cxcl12*-dsRED; *Cxcr4*-EGFP mouse antrum without (control antrum) and after MNU treatment (MNU-treated), and *Mist1*-CreERT; *Cxcl12*-dsRED; *Cxcr4*-EGFP; *Apc*^flox/flox^ mice 6 weeks after TAM induction (*Apc*^flox/flox^). Cxcl12^+^ and Cxcr4^+^ areas were measured in (D). A total of 30 high power fields (HPF) from three mice were analyzed. (**E**) 3D reconstructed images of *Mist1*-CreERT; *Apc*^flox/flox^; *Cxcr4*-EGFP; *Cxcl12*-dsRED mouse antrum 60 days after tamoxifen stained with DAPI. (**F**) GFP and Ki67 staining of Cxcr*4*-EGFP; *Cxcl12*^flox/flox^ (control) mice and *Tie2*-Cre; *Cxcr4*-EGFP; *Cxcl12*^flox/flox^ mice 40 weeks after the start of 5 cycles of MNU treatment. Cxcr4^+^ and Ki67^+^ epithelial cell ratio of *Cxcr4*-EGFP; *Cxcl12*^flox/flox^ (control) mice and *Tie2*-Cre; *Cxcr4*-EGFP; *Cxcl12*^flox/flox^ mice were quantified. The total 1500 cells from three mice are analyzed. (**G**) Macroscopic antral tumor size was measured in *Cxcl12*^flox/flox^ (control, *N* = 13) mice and *Tie2*-Cre; *Cxcl12*^flox/flox^ mice (*N* = 7) 40 weeks after the start of 5 cycles of MNU treatment. (**H**) Relative mRNA expression/*Gapdh* of the indicated genes from the MNU-induced tumor tissues in *Cxcl12*^flox/flox^ mice (Ctr) and *Tie2*-Cre; *Cxcl12*^flox/flox^ mice (Tie2).

We next investigated the contribution of the Cxcl12/Cxcr4 axis to antral gastric tumorigenesis using two different mouse models: conditional *Apc* knockout in *Mist1*^+^ cells and MNU-induced antral tumorigenesis. While Cxcr4^+^ epithelial cells are normally at the gland base surrounded by Cxcl12^+^ endothelial cells, there was marked expansion of Cxcr4^+^ epithelial cells to within the antral tumor (Figure 5A–5D). *Cxcr4* gene expression is upregulated in tumors than in normal antrum. In addition, there was a corresponding expansion of Cxcl12^+^ stromal cells. Tissue decolorization and 3D reconstitution successfully emphasized remarkable expansion of Cxcl12/Cxcr4 expressing cells within antral tumor (Figure 5E). Interestingly, there are strong *Cxcr4*-GFP expressing clusters within the tumor, suggesting the clonal expansion of Cxcr4^+^ cells in dysplastic glands.

To elucidate the functional role of the Cxcl12/Cxcr4 axis in antral tumorigenesis, we generated *Tie2*-Cre; *Cxcl12*^flox/flox^ mice with targeted deletion of *Cxcl12* in endothelial cells, and used these animals in the MNU tumor model. In untreated mice at baseline, the expression of Cxcr4 and Ki67 in antral epithelial cells of *Tie2*-Cre; *Cxcr4*-EGFP; *Cxcl12*^flox/flox^ mice and *Cxcr4*-EGFP; *Cxcl12*^flox/flox^ mice (control littermates) was comparable (Supplementary Figure 5A). However, while MNU treatment caused marked proliferation (increased Ki67^+^ cells) and expansion of Cxcr4^+^ epithelial cells in control mice, MNU-treated *Tie2*-Cre; *Cxcr4*-EGFP; *Cxcl12*^flox/flox^ mice showed much smaller changes, with significantly decreased Cxcr4^+^ epithelial cells and Ki67^+^ cells compared to MNU treated controls (Figure 5F).

To assess the contribution of Cxcr4^+^ ILCs in antral tumor development, we treated MNU-treated mice with an anti-CD90.2 antibody. While treatment with the anti-CD90.2 antibody efficiently depleted CD90.2^+^ ILCs, MNU-induced tumor development was not inhibited in this protocol (Supplementary Figure 5B–5D). Thus, these data suggest that the expansion of the Cxcl12^+^ endothelium may contribute to antral tumorigenesis in this model predominantly through activation of Cxcr4^+^ epithelial stem/progenitors, rather than through regulation of ILCs.

The macroscopic tumor size was significantly smaller in the MNU-treated *Tie2*-Cre; *Cxcr4*-EGFP; *Cxcl12*^flox/flox^ mice compared to controls (Figure 5G). RT-PCR analysis revealed that deletion of Cxcl12 in the endothelium downregulated gene expression of *Cxcr4* as well as *Mist1*, while *Cckbr* was upregulated in *Tie2*-Cre; *Cxcl12*^flox/flox^ mice compared to control mice (Figure 5H). *Lgr5* gene expression was not altered by conditional Cxcl12 knockout. These results suggest that knockout of Cxcl12 in the endothelium inhibited expansion of the *Mist1*^+^Cxcr4^+^ cell population, but the loss of *Mist1*^+^ cells may have been partially compensated by CCK2R^+^ stem cell expansion.

### Pharmacological blockade of Cxcr4 inhibits antral tumor growth

**Figure 6:**
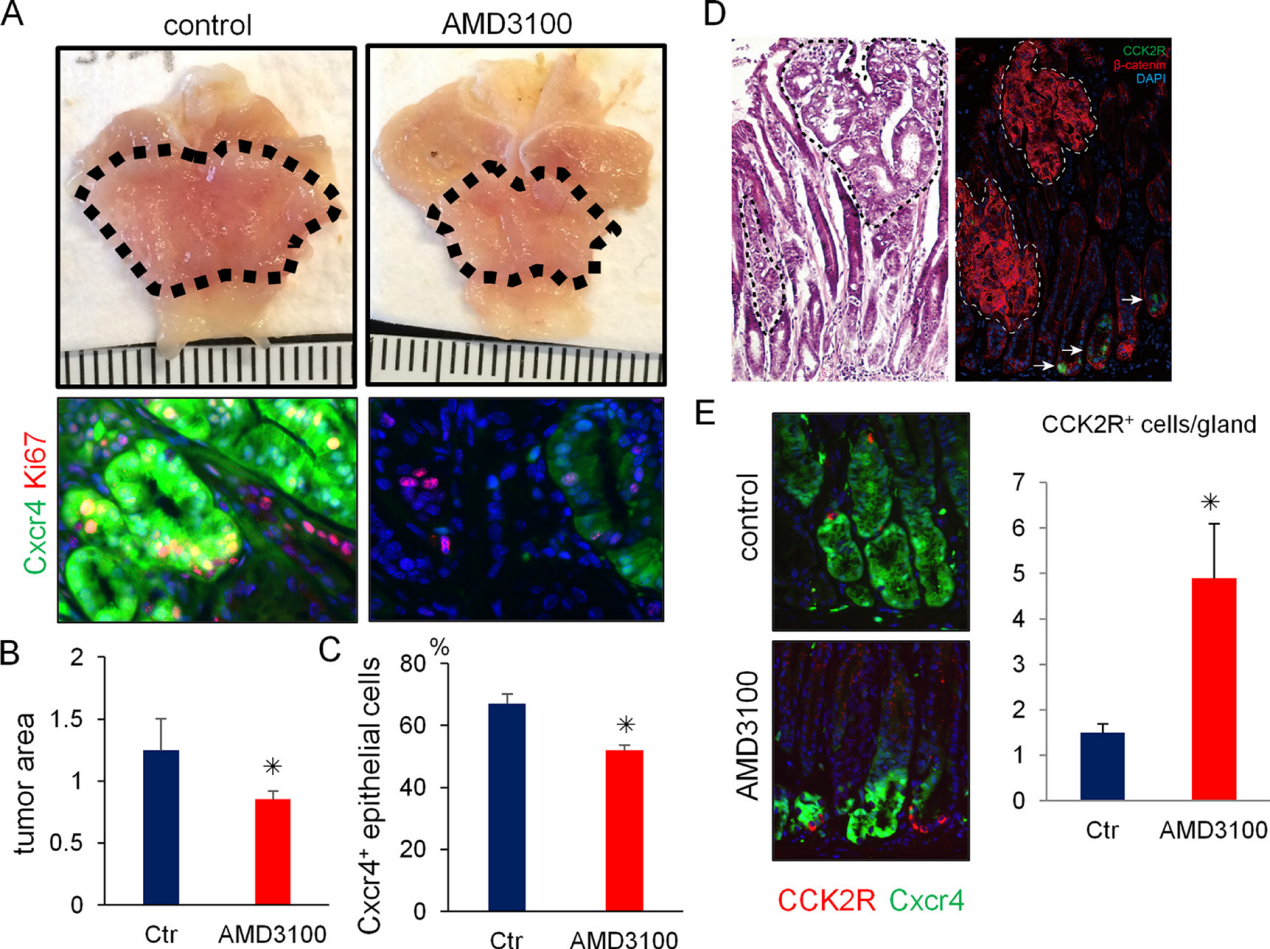
Pharmacological blockade of Cxcr4 inhibits antral tumor growth (**A**–**C**) Gross picture (A, top) and immunofluorescence images (A, bottom) of GFP (green) and Ki67 (red) in *Mist1*-CreERT; *Cxcr4*-EGFP; *Apc*^flox/flox^ mice 6 weeks after TAM induction with or without 2 weeks AMD3100 treatment. The dashed-line indicates tumor area. Macroscopic antral tumor area was quantified in (B) (*N* = 4 /group). The percentage of Cxcr4^+^ epithelial cells per total Ki67^+^ cells was quantified in (C). A total of 1500 cells from three mice were analyzed. (**D**) H&E staining and immunofluorescence of CCK2R (green) and β-catenin (red) in *Mist1*-CreERT2; *Apc*^flox/flox^ mice 6 weeks after TAM induction. (**E**) Immunofluorescence of GFP (green) and CCK2R (red) in *Mist1*-CreERT2; *Apc*^flox/flox^ mice 6 weeks after TAM induction with or without AMD3100 treatment. CCK2R^+^ cells/gland were counted. A total 150 glands from three mice were analyzed.

Finally, to test the possible therapeutic utility of pharmacologic inhibition of the Cxcl12/Cxcr4 axis on antral tumor development, we treated *Mist1*-CreERT; *Cxcr4*-EGFP; *Apc*^flox/flox^ mice with AMD3100, a specific inhibitor of CXCR4. AMD3100 significantly reduced the number of Cxcr4^+^ epithelial cells in the gastric antrum and decreased macroscopic tumor size (Figure 6A–6C). In the *Mist1*-CreERT; *Apc*^flox/flox^ mice, most dysplastic cells demonstrated nuclear translocation of β-catenin. However, CCK2R^+^ cells in these murine tumors did not show nuclear expression of β-catenin (Figure 6D). Thus, the majority of *Apc*-deleted *Mist1*^+^ cells do not interconvert to CCK2R^+^ cells during this rapid tumor formation. Furthermore, the numbers of CCK2R^+^ cells were increased when mice were treated with AMD-3100 (Figure 6E), suggesting again that in this tumorigenic setting, CCK2R^+^ cells may behave as a compensatory lineage, distinct from *Mist1*^+^ cells.

## DISCUSSION

In the current study, we identified *Mist1*^+^ long-lived progenitor cells in the gastric antrum that are distinct from other reported gastric stem cell populations, including *Lgr5*^+^ cells and CCK2R^+^ cells. In addition, we have shown that antral *Mist1*^+^ cells can serve as an origin of gastric tumors in a different way from the corpus *Mist1*^+^ stem cells. *Mist1*^+^ progenitors overlap in the antrum with Cxcr4^+^ antral stem/progenitor cells, which appear to be supported by Cxcl12 secreted from endothelial cells within a perivascular niche. Activation of Cxcl12/Cxcr4 signaling appears to be needed for antral tumor growth.

Earlier studies using electron microscopy and autoradiography suggested that antral stem cells with an undifferentiated, granule-free appearance reside within the isthmus, and supply daughter cells bidirectionally towards the top and the base of the glands [24, 25]. Several markers have been reported for actively cycling antral stem cells, but until now clear relationship between these markers has not been well established [10–12]. In the paper describing *Villin*^+^ progenitors [14], the authors used *Villin*-LacZ and *Villin*-Cre mice and found rare *Villin*^+^ cells in the isthmus of antral glands which can show traced glands only when mice are treated with interferon (INF). The phenotype of INF-dependent tracing definitely suggests that *Villin*^+^ cells are not stem or progenitor cells in normal state and distinct from *Mist1*^+^ cells. Sox2 was initially reported to be expressed in rare stem cells within the corpus and antrum isthmus in the adult stomach [11]. More recently Sox2 was found to be expressed in broader transit-amplifying cells in the antral isthmus, which contribute to tumor development in the antrum with loss of *Apc* [21]. The enhancer element eR1 is recently reported corpus/antral stem and progenitor cell marker [13], but the expression is scattered and its biology and contribution to cancers are not fully determined. *Axin2* is also expressed in both broad isthmus progenitor cells and basal *Lgr5*^+^ cells, and likely overlaps with eR1 or other markers. Finally, CCK2R^+^ antral stem cells which were identified by our group [12] reside at +4 position and appear to show quite close proximity to *Mist1*^+^ antral cells. Our current findings using fluorescent imaging and FACS analysis failed to demonstrate the evident overlap between *Mist1* and other markers. Nonetheless, we do not exclude the possibility that some of reported stem/progenitor cell markers (e.g., eR1, Sox2, or *Axin2*) may contain *Mist1*^+^ cells when using reported CreERT lines. It should be noted that *Mist1* expression is more restricted to the isthmus region than other markers, however, it may be possible that *Mist1* marks heterogeneous isthmus progenitors that are long-lived.

We show here that *Mist1*^+^ cells can serve as the cell-of-origin for antral gastric tumors. It has long been suspected that cancer arises from genetic alterations in stem cells, given their longevity and capacity for self-renewal [26]. Indeed, *Lgr5*^+^ and Sox2^+^ cells can also give rise to dysplasia or tumors with loss of the *Apc* gene [10, 21]. We have shown here that relatively quiescent *Mist1*^+^ cells can give rise to cancer after exposure to the carcinogen (MNU) or loss of *Apc*. In the distal stomach, intestinal-type gastric cancer is the more common histologic type, and is typically associated with activation of the Wnt/β-catenin signaling pathway [10, 27]. Indeed, the frequency of *Apc* mutation in human intestinal-type gastric cancer is significantly higher than that in diffuse-type [28]. The fact that *Apc* mutation models in the stomach resulted only in antral tumors without corpus tumor may suggest that the susceptibility to cancer in response to certain oncogenes and carcinogens is different between the antrum and corpus. In humans, MSI^+^ cancers are more common in antral cancers, while *Tp53*-mutated CIN^+^ tumors are found more often in proximal cancers. Thus, it may be crucial to understand the molecular mechanism in both antral and corpus carcinogenesis separately.

In previous studies, we found that the development of diffuse-type corpus cancer was highly dependent on an endothelial niche, with Cxcl12 signaling from endothelial cells to Cxcr4-expressing ILCs, which then activate *Mist1*^+^ corpus stem cells through Wnt5a secretion. In the current study, we observed a similar juxtaposition of Cxcl12-expressing endothelial cells adjacent to *Mist1*^+^ antral cells. In addition, we previously reported that Cxcl12/Cxcr4 signaling is involved in activation and recruitment of cancer-associated fibroblasts during gastric carcinogenesis, and that aberrant expression of Cxcl12 accelerates gastric cancer development [22, 29]. Here, we show clear Cxcr4 expression in the *Mist1*^+^ antral cells, and in response to carcinogen exposure, there was a marked expansion of Cxcr4^+^ epithelial cells that eventually encompassed almost the entire gland. Thus, Cxcr4 signal may broadly affect gastric carcinogenesis through multiple mechanisms.

Interestingly, while Cxcr4 inhibition leads to suppression of *Mist1*-derived tumor growth, there is compensatory activation of the CCK2R lineage, which we showed was negative for Cxcr4 expression. Thus, in the absence of Cxcl12/Cxcr4 signaling and reduction in the *Mist1* lineage, there is an upregulation of CCK2R^+^ cells (but interestingly not *Lgr5*^+^ cells), suggesting that other stem cell populations can compensate for loss of the *Mist1* lineage. Given this observation, it may be interesting to consider simultaneous blockade of the Cxcr4 axis and inhibition of CCK2R^+^ cells may achieve better tumor suppression.

*MIST1* expression in human gastric antrum was recently described [30], and epithelial Cxcr4 expression in human gastric cancer tissue has been also reported [31, 32]. In addition, a meta-analysis reported that Cxcr4 expression in primary human gastric cancer tissues was positively associated with tumor progression and disease prognosis, including vascular invasion [33]. Thus, we believe that Cxcr4^+^ epithelial cells, which include the *Mist1*^+^ antral progenitors, contribute to human gastric cancer progression, and that the Cxcr4/Cxcl12 axis may still be a promising therapeutic target against broad spectrum of gastric cancers. While targeting the endothelium using antibodies to vascular endothelial growth factor receptor 2 (VEGFR-2) has shown benefit in some tumors in clinical trials [34, 35], the role of Cxcl12 secretion in the response has not been explored. The current study provides further support for the existence of Cxcl12 endothelium as a representing key niche for gastrointestinal stem cells.

## MATERIALS AND METHODS

### Mice

*Mist1*-CreERT mice [36] and *Cxcl12*-dsRED mice [37] were described previously. *Cxcr4*-EGFP mice were provided from Richard J. Miller (North-western University Medical School). LSL-*Kras*^G12D^ and LSL-*Trp53*^R172H^ mice were provided by Dr. Kenneth Olive (Columbia University). *Cckbr*-GFP BAC transgenic mice were purchased from MMRRC (GENSAT project [38]). *Lgr5*-DTR-GFP mice were provided by Genentech. *Apc*^flox/flox^ mice were obtained from the National Cancer Institute. *Lgr5-*EGFP-IRES-CreERT*, Sox2*-EGFP, *R26*-mTmG, *R26*-TdTomato, *R26-*LacZ*, Cxcl12*^flox/flox^, and *Tie2*-Cre mice were purchased from the Jackson Laboratory. FucciG1 mice were obtained from RIKEN BRC. Cre recombinase was activated by oral administration of tamoxifen (TAM, Sigma, 3 mg/0.2 ml corn oil). All animal studies and procedures were approved by the ethics committees at Columbia University and the University of Tokyo. All mice were bred under specific pathogen free conditions. Comparisons were made with age- and sex- matched control animals.

### Treatment

For *Lgr5*^+^ cell ablation, diphtheria toxin (DT, Sigma) was administered intraperitoneally (20mg/kg). N-nitroso-N-methylurea (MNU, Sigma) was dissolved in distilled water at a concentration of 240 ppm and administered in drinking water. Mice (8-week-old) were given drinking water containing MNU on alternate weeks for five cycles [12]. Mice were analyzed 50 weeks after the beginning of the MNU treatment. Mice were treated with CD90.2 mAb (30H12) (BioXcell) intraperitoneally at a dose of 250 µg/mouse for 4 weeks. AMD3100 (Tocris) was administered at a dose of 5mg/kg/day by implanted subcutaneous osmotic pumps (Alzet 2004) for 2 weeks.

### Statistical analysis

The difference between the means was compared by either Student’s *t*-test or the Wilcoxon test. *p* values < 0.05 were considered to indicate statistical significance. Other detailed information is described in Supplementary methods.

## SUPPLEMENTARY MATERIALS FIGURES



## Abbreviations

ILCs: innate lymphoid cells
MNU: N-nitroso-N-methylurea
TAM: tamoxifen
DT: diphtheria toxin
BrdU: bromodeoxyuridine
