# Supplementary material for: CXCR4-expressing Mist1+ progenitors in the gastric antrum contribute to gastric cancer development
Source: Oncotarget. 2017 Nov 10;8(67):111012–25. doi: 10.18632/oncotarget.22451 (PMC5762301; doi:10.18632/oncotarget.22451)
Supplement: Supplementary file 1 [file oncotarget-08-111012-s001.pdf]

CXCR4-expressing *Mist1*+ progenitors in the gastric antrum contribute to gastric cancer development

SUPPLEMENTARY MATERIALS

SUPPLEMENTAL EXPERIMENTAL PROCEDURES

Gland isolation and *in vitro* culture system

Gland isolation and organoid culture were performed as described previously [1-3]. To obtain single cells, glands were dissociated with TrypLE express (Invitrogen) including 1 mg/ml DNase I (Roche) for 10 minutes. Dissociated cells were passed through a 20 µm cell strainer and washed with 2% FBS/PBS. Viable epithelial single cells were gated by forward scatter, side scatter and a pulse-width parameter, and negative staining for propidium iodide. For staining, cell suspensions were incubated with conjugated monoclonal antibodies against CD45 (BioLegend) and lineage-cocktail antibodies (BioLegend). Sorted cells were collected and embedded in Matrigel, followed by seeding on a 48-well plates (100-3000 singlets per well). The images of organoids were taken using fluorescent microscopy (Nikon, TE2000-U) or 2-photon microscopy (Nikon, A1RMP).

Histopathologic analysis

Tissues were fixed in 10% formalin or 4% paraformaldehyde overnight, embedded in paraffin or OCT respectively and cut into 5 µm sections. Immunohistochemistry and immunofluorescence were performed as described previously [3, 4]. The following primary antibodies were used; Mist1 (1:500, provided by Dr. Konieczny), TFF1 (1:100, Santa Cruz), Gastrin (1:200, Santa Cruz), Dclk1 (1:200, Abcam), Somatostatin (1:200, Abcam), CCK2R (1:200, Santa Cruz), BrdU (1:500, Dako), GFP (1:400, Invitrogen), β-catenin (1:500, BD biosciences), Ki67 (1:100, Abcam), phalloidin (1:200, Invitrogen), CD31 (1:200, Dako), podoplanin (1:200, R&D systems), α-SMA (1:500, Abcam), CD90.2 (1:200, BD biosciences), Axin2 (1:100, Abcam), and E-cadherin (1:100, Cell Signaling Technology). Primary antibodies were incubated overnight, and subsequently, the sections were incubated with biotinylated secondary antibodies (Vectastatin ABC kit; Vector Laboratories) for 30 minutes, followed by incubation with avidin-coupled peroxidase (Vector Laboratories). Diaminobenzidine (DAB; Dako) was used as the chromogen and the slides were counterstained with hematoxylin. For immunofluorescence, the slides were incubated with Alexa flour 488 or 555 secondary antibodies (Invitrogen) and counterstained with 4′, 6-diamidino-2-phenylindole (Vector Laboratories). RNAScope-based in situ hybridization was performed on paraffin-embedded sections using the RNAScope 2.0 kit (Advanced Cell Diagnostics).

In situ hybridization

Mouse stomach was dissected, fixed with G-Fix (Genostaff), embedded in paraffin on CT-Pro20 (Genostaff) using G-Nox (Genostaff) as a less toxic organic solvent for Xylene, and sectioned at 5um. In situ hybridization was performed with the ISH Reagent Kit (Genostaff) according to manufacturer's instructions. Tissue sections were deparaffinized with G-Nox, and rehydrated through an ethanol series and PBS. The sections were fixed with 10% NBF (10%Formalin in PBS) for 15 min at room temperature and washed in PBS, treated with 4 ug/ml Proteinase K (Wako Pure Chemical Industries) in PBS for 10 min at 37 C and washed in PBS, re-fixed with 10% NBF for 15 min and washed in PBS, placed in 0.2N HCl for 10 min and washed in PBS, and placed within a coplin jar containing 1x G-Wash (Genostaff), equal to 1x SSC. Hybridization was performed with probes at concentrations of 300 ng/ml in G-Hybo-L (Genostaff) for 16 hours at 60 C. After hybridization, the sections were washed in 1x G-Wash for 10 min at 60 C, 50% formamide in 1x G-Wash for 10 min at 60 C. Then the sections were washed twice in 1x G-Wash for 10 min at 60 C, twice in 0.1x G-Wash for 10 min at 60 C, and twice in TBST (0.1% Tween 20 in TBS). After treatment with 1x G-Block (Genostaff) for 15 min, the sections were incubated with anti-DIG AP conjugate (Roche Diagnostics) diluted 1:2000 with x50 G-Block (Genostaff) in TBST for 1 hour. The sections were washed twice in TBST and then incubated in 100 mM NaCl, 50 mM MgCl2, 0.1% Tween 20, 100mM Tris-HCl, pH 9.5. Coloring reactions were performed with NBT/BCIP solution (Sigma-Aldrich) overnight and then washed in PBS. The sections were counterstained with Kernechtrot stain solution (Muto Pure Chemicals), and mounted with G-Mount (Genostaff). The probe sequence is as

follows: GGAGAACCCTGGTACCCGAAAGAACTAGATAATAGATACACTAGGCCTGGGGGTGTTGGTCTCCTTATCCTGGTGAGACTTGATTTGTATAGCTTTCCAGTCCTGGTCTGGTCCCTTCCTAGATCGTCCGTAGGTCAGGTAGAGACTGGGGGGCCTGTGTGTCTGTTACTGGGGACGGCTACTGGGAAAGGTCAGG GAATTTGCCAGTGTGAGCGGAGGCCAGGCATGACTCCAAGCTGGAAAGACAAAGCCCAGATATCTACCTTGCTATGGGGAGCAAAGGCCTCTGCCGACAGACAGACCTACAAACCCCATGGCTGGCTTCCGTATGAGCCAAGGCCAGAGCGGGGCTCCTTTTGTCCAGCCAAACCTCTGCCCTAATGCCCGGCTGCCCTAATGCCCAGGGCTGTCCAGGGTCTCTGTTTGGCGGGACCTCAGCCTCCTGGCTCTTTGTCCCCCCTTTTGTATACCCCTGCTTCCTAGGGGGTGACATGGGCCTAGCTGTCCAGTTCTTTACTGGTGACAGGCAGGACCTACATGTCCCTTGGCCACATTACAAAGGATCTTTAGAGACTCCGGTCCCGTTAGGAGTCTAGGAAGCCCTA.

Quantitative analysis of mRNA expression

Total RNA was extracted from snap-frozen gastric tissues in dry ice with NucleoSpin RNA II kit (Clontech) and cDNA was synthesized by Superscript III First-strand Synthesis System (Invitrogen). Expression levels of indicated genes were determined by real-time PCR using SYBR Green (Thermo Fisher) and 7300 Real Time PCR System (Applied Biosystems). Gapdh mRNA was used as internal control. The primer sequence used are available on request.

Tissue decolorization

Stomach tissues were fixed in 4% paraformaldehyde at 4°C for 24 hours. The tissues were washed in PBS and incubated in DAPI solution in PBS for 24 hours at 4°C. Then tissues were incubated in pretreatment solution for 24 hours at room temperature. The samples were then transferred to the final clearing solution (LUCID-A, modified from original LUCID [5]) at 37°C, and were stored at 4 °C until the scheduled multi-photon microscopic observation. All washes and incubations were done in a light-resistant container by constant and gentle shaking. A Nikon A1RMP two-photon microscope (Nikon, Tokyo, Japan) was used for imaging. The images were processed using NIS-Elements C and AR (Nikon).

REFERENCES

1. Barker N, Huch M, Kujala P, van de Wetering M, Snippert HJ, van Es JH, Sato T, Stange DE, Begthel H, van den Born M, Danenberg E, van den Brink S, Korving J, et al. Lgr5(+ve) stem cells drive self-renewal in the stomach and build long-lived gastric units in vitro. Cell Stem Cell. 2010; 6:25–36.
2. Zhao CM, Hayakawa Y, Kodama Y, Muthupalani S, Westphalen CB, Andersen GT, Flatberg A, Johannessen H, Friedman RA, Renz BW, Sandvik AK, Beisvag V, Tomita H, et al. Denervation suppresses gastric tumorigenesis. Sci Transl Med. 2014; 6:250ra115.
3. Hayakawa Y, Jin G, Wang H, Chen X, Westphalen CB, Asfaha S, Renz BW, Ariyama H, Dubeykovskaya ZA, Takemoto Y, Lee Y, Muley A, Tailor Y, et al. CCK2R identifies and regulates gastric antral stem cell states and carcinogenesis. Gut. 2015; 64:544–53.
4. Hayakawa Y, Hirata Y, Nakagawa H, Sakamoto K, Hikiba Y, Kinoshita H, Nakata W, Takahashi R, Tateishi K, Tada M, Akanuma M, Yoshida H, Takeda K, et al. Apoptosis signal-regulating kinase 1 and cyclin D1 compose a positive feedback loop contributing to tumor growth in gastric cancer. Proc Natl Acad Sci U S A. 2011; 108:780–5.
5. Sekitani T, Yokota T, Kuribara K, Kaltenbrunner M, Fukushima T, Inoue Y, Sekino M, Isoyama T, Abe Y, Onodera H, Someya T. Ultraflexible organic amplifier with biocompatible gel electrodes. Nat Commun. 2016; 7:11425.

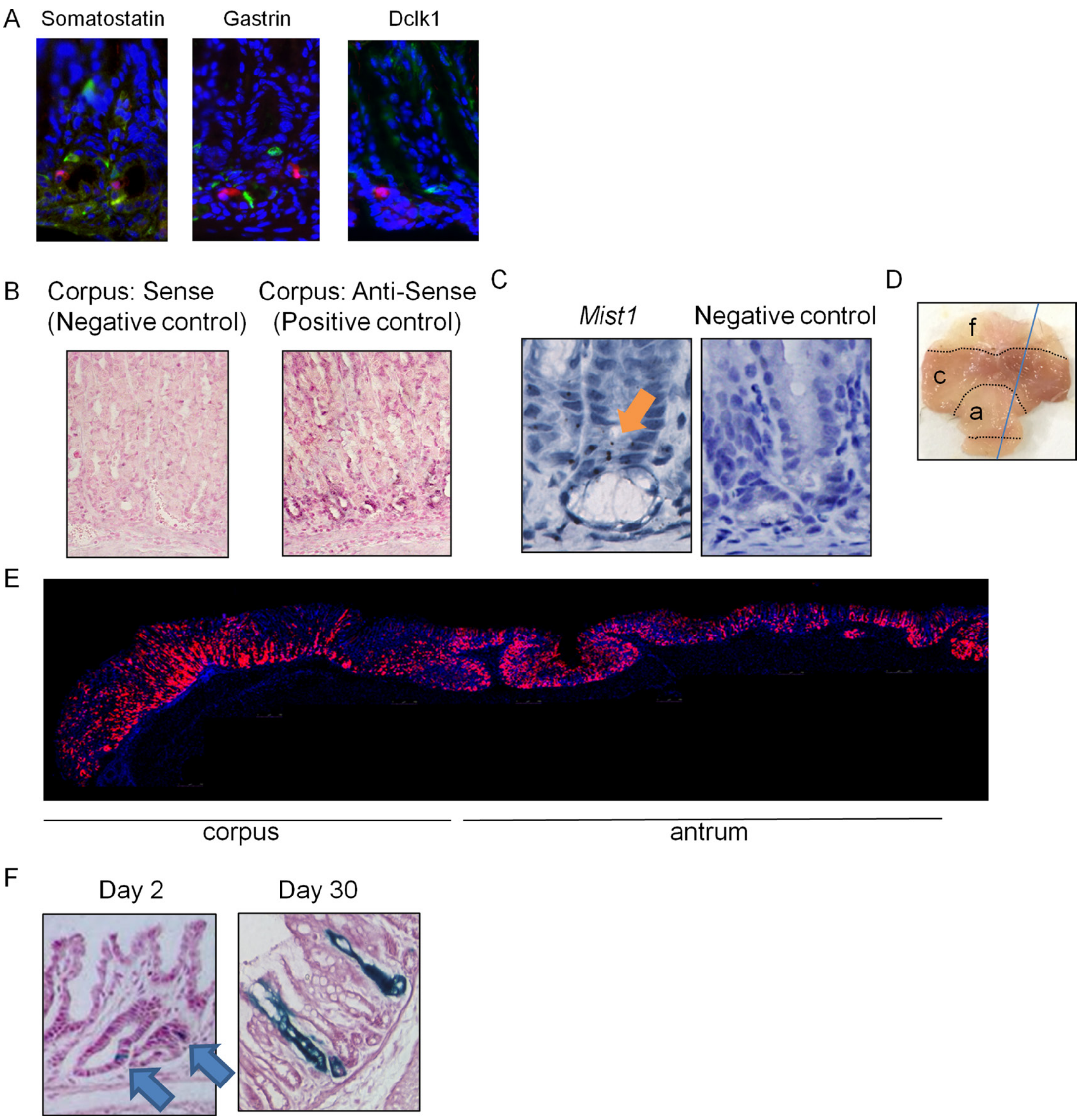

Supplementary Figure 1: *Mist1* expression in the antrum. (**A**) Immunostaining (green) of the indicated markers in *Mist1*-CreERT; *R26*-TdTomato mice 1 day after tamoxifen. (**B**) In situ hybridization of *Mist1* in the corpus glands with the conventional method. (**C**) In situ hybridization of *Mist1* in the antral glands using RNAscope method. (**D**) Anatomy of the mouse stomach. f; forestomach, c; corpus, and a; antrum. (**E**-**F**) Lineage tracing in *Mist1*-CreERT; *R26*-TdTomato (E) and *Mist1*-CreERT; *R26*-LacZ (F) mouse antrum.

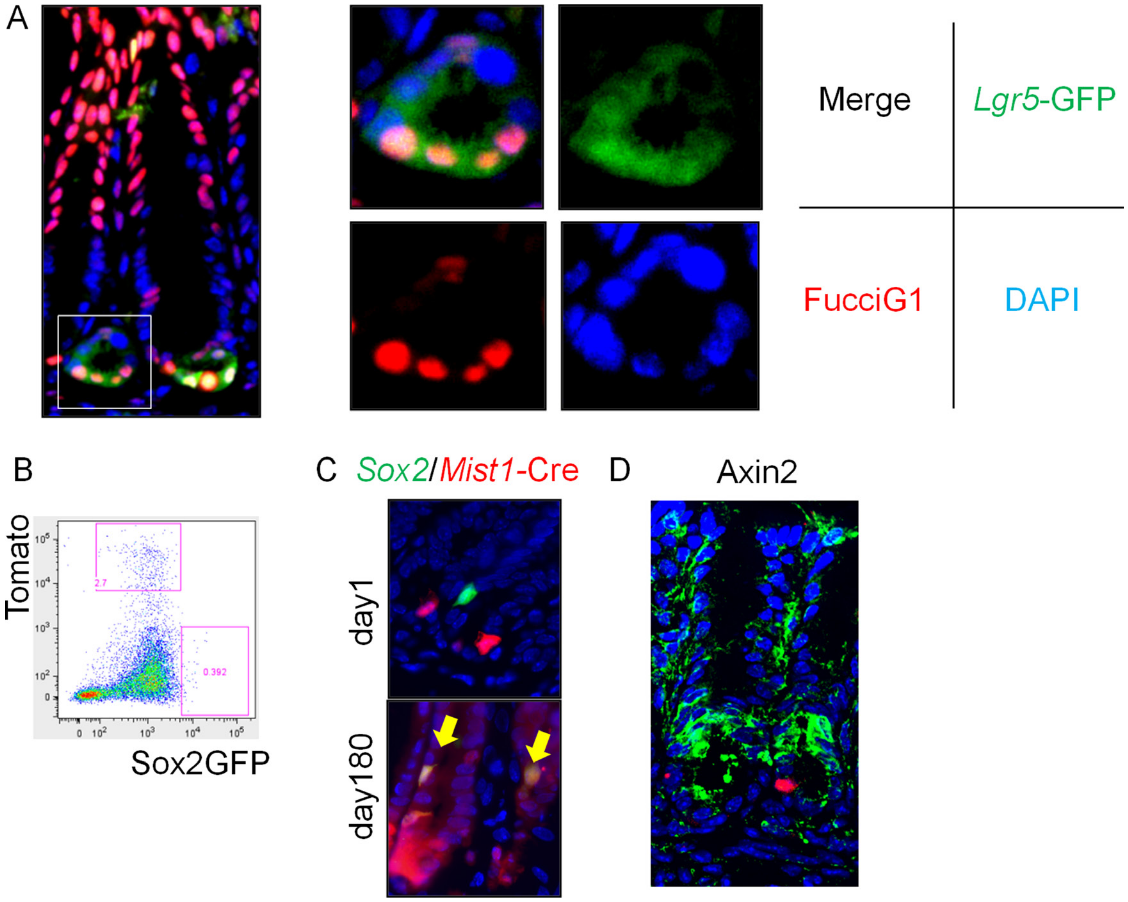

Supplementary Figure 2: Cell cycle status in gastric *Lgr5*+ cells. (**A**) Antral glands of *Lgr5*-EGFP-IRES-CreERT (green); FucciG1 (red) mice stained with DAPI (blue). Area shown by white box in left panel is enlarged in right panels with single and merged color images. (**B**) FACS plot of *Mist1*-CreERT; *Sox2*-GFP; *R26*-TdTomato antrum 1 day after tamoxifen. (**C**) Day 1 and 180 images of *Mist1*-CreERT; Sox2-GFP; *R26*-TdTomato antrum. Arrow indicates double positive cells. (**D**) Immunostaining (green) of Axin2 in *Mist1*-CreERT; *R26*-TdTomato mice 1 day after tamoxifen.

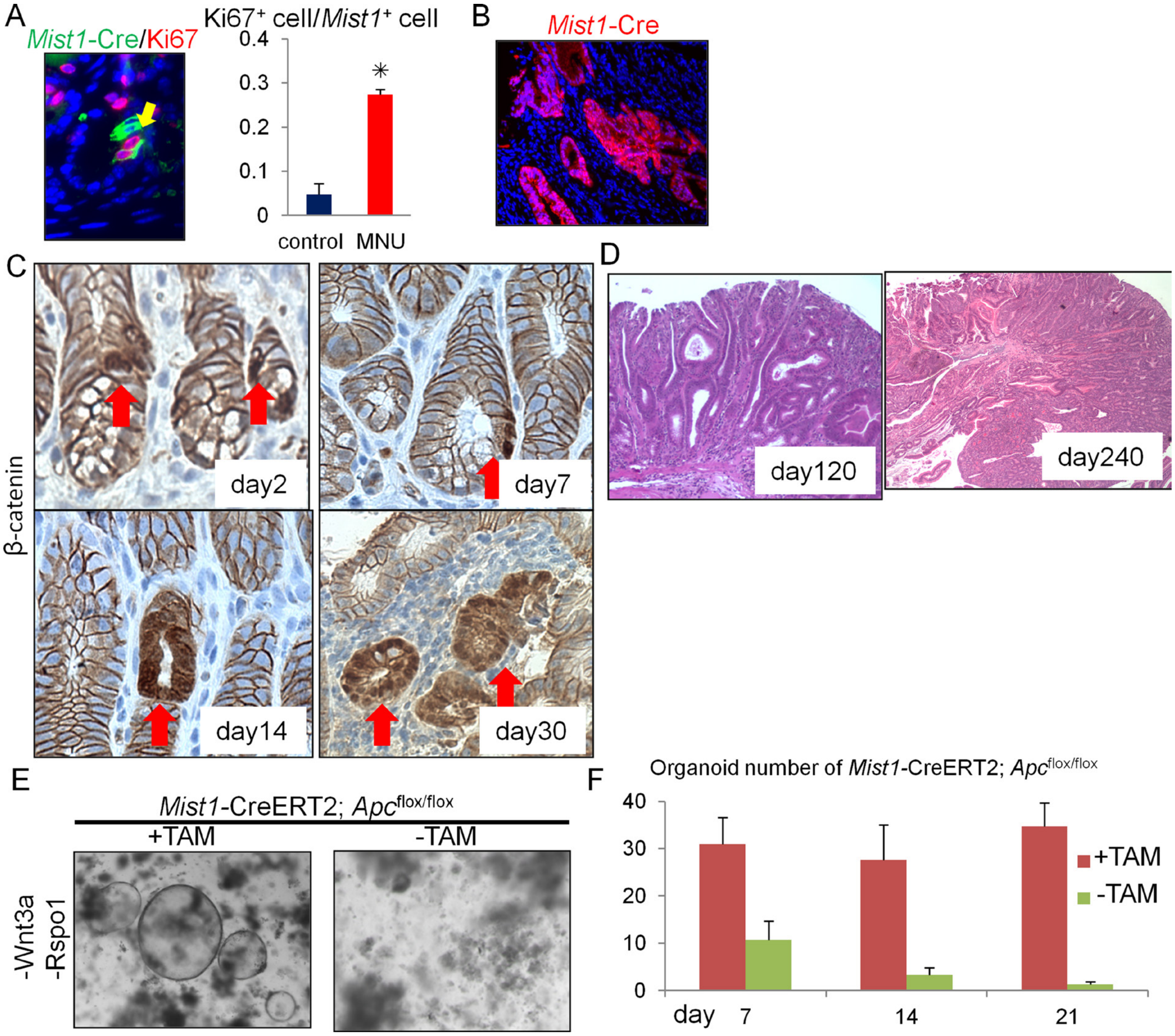

Supplementary Figure 3: Antral tumor development derived from *Mist1*$^{+}$ stem cells. (**A**) (left) Immunofluorescence of GFP (green) and Ki67 (red) in *Mist1*-CreERT; *R26*-mTmG mice after 5 cycles of MNU treatment. Mice were sacrificed 1 day after tamoxifen. Arrow indicates double-positive cell. (right) Ki67^{+} cell ratio of *Mist1*$^{+}$ cells in *Mist1*-CreERT; *R26*-mTmG mice 1 day after TAM induction. Mice were treated with or without 5 cycles of MNU, then TAM was given. A total of 300 cells from three mice were analyzed. (**B**) Lineage tracing in MNU-treated *Mist1*-CreERT; *R26*-TdTomato mouse antral tumor. TAM was given before starting MNU treatment. (**C**) β-catenin staining on *Mist1*-CreERT; *Apc*$^{flox/flox}$ mice on days 2, 7, 14, 30 after TAM induction. The arrows indicate the nuclear β-catenin+ cells. (**D**) H&E images of *Mist1*-CreERT; *Apc*$^{flox/flox}$ mice at indicated time points. (**E–F**) Representative images and numbers of *Mist1*-CreERT; *Apc*$^{flox/flox}$ mouse antrum-derived organoids with or without 4-OH tamoxifen. Wnt3a and R-spondin1 were removed from culture media. Supplementary Figure 3. Cxcl12^{+} cells are not lymphatic vessels nor fibroblasts.Immunostaining of Cxcl12-dsRED^{+} cells with podoplanin and α-SMA (green).

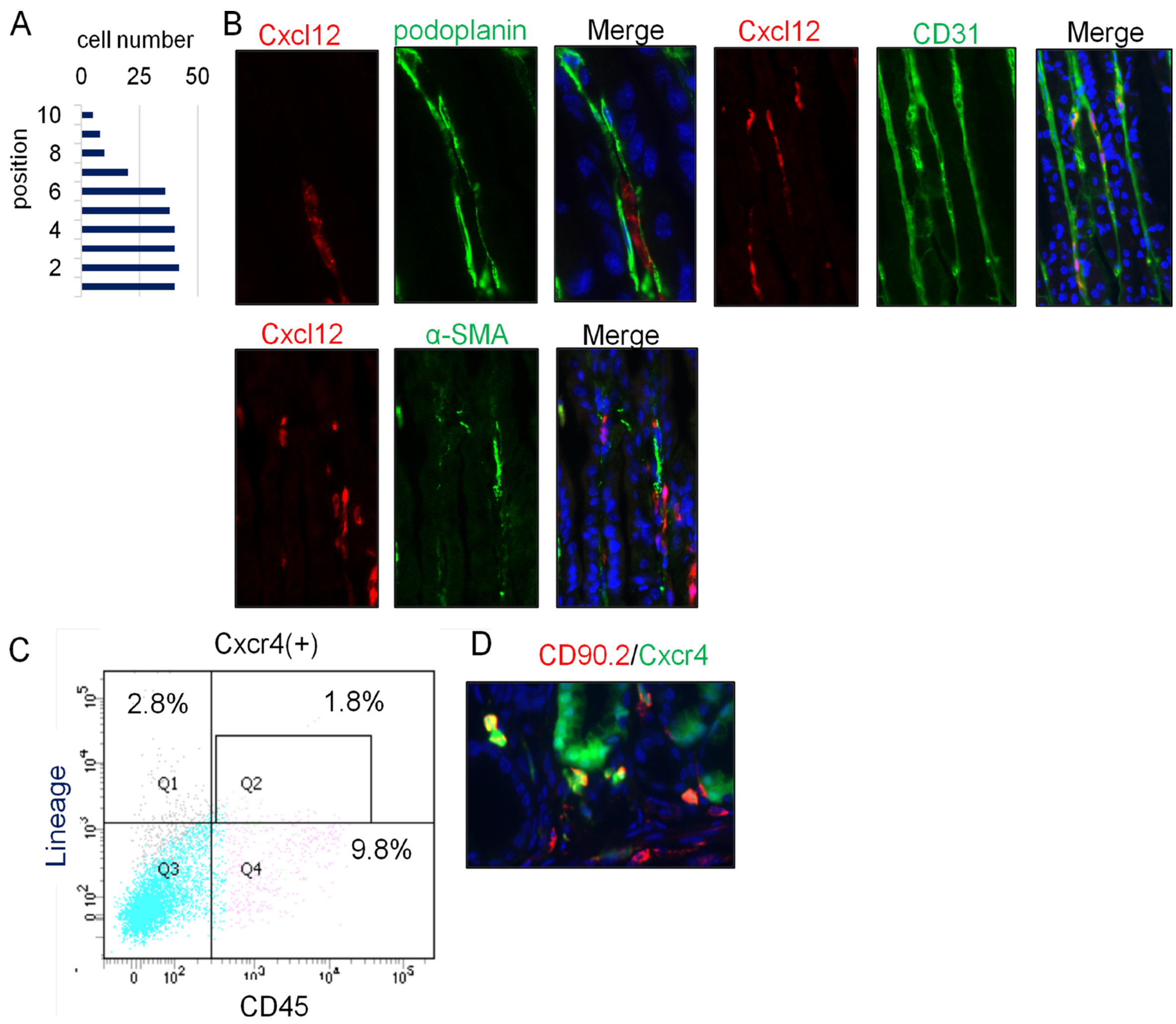

Supplementary Figure 4: Characterization of Cxcl12/Cxcr4 niche in the antrum. (**A**) Quantification of $Cxcr4^+$ epithelial cell position in the antral glands. The total 300 cells from three mice are analyzed. (**B**) Immunofluorescence staining of CD31, podoplanin, and α-SMA (green) in *Cxcl12*-dsRED mice. (**C**) FACS plot of antral GFP^+ cells from *Cxcr4*-EGFP mice gated by $CD45^+$ and Lin^-. (**D**) CD90.2 (red) staining of *Cxcr4*-$EGFP^+$ cells in the antrum.

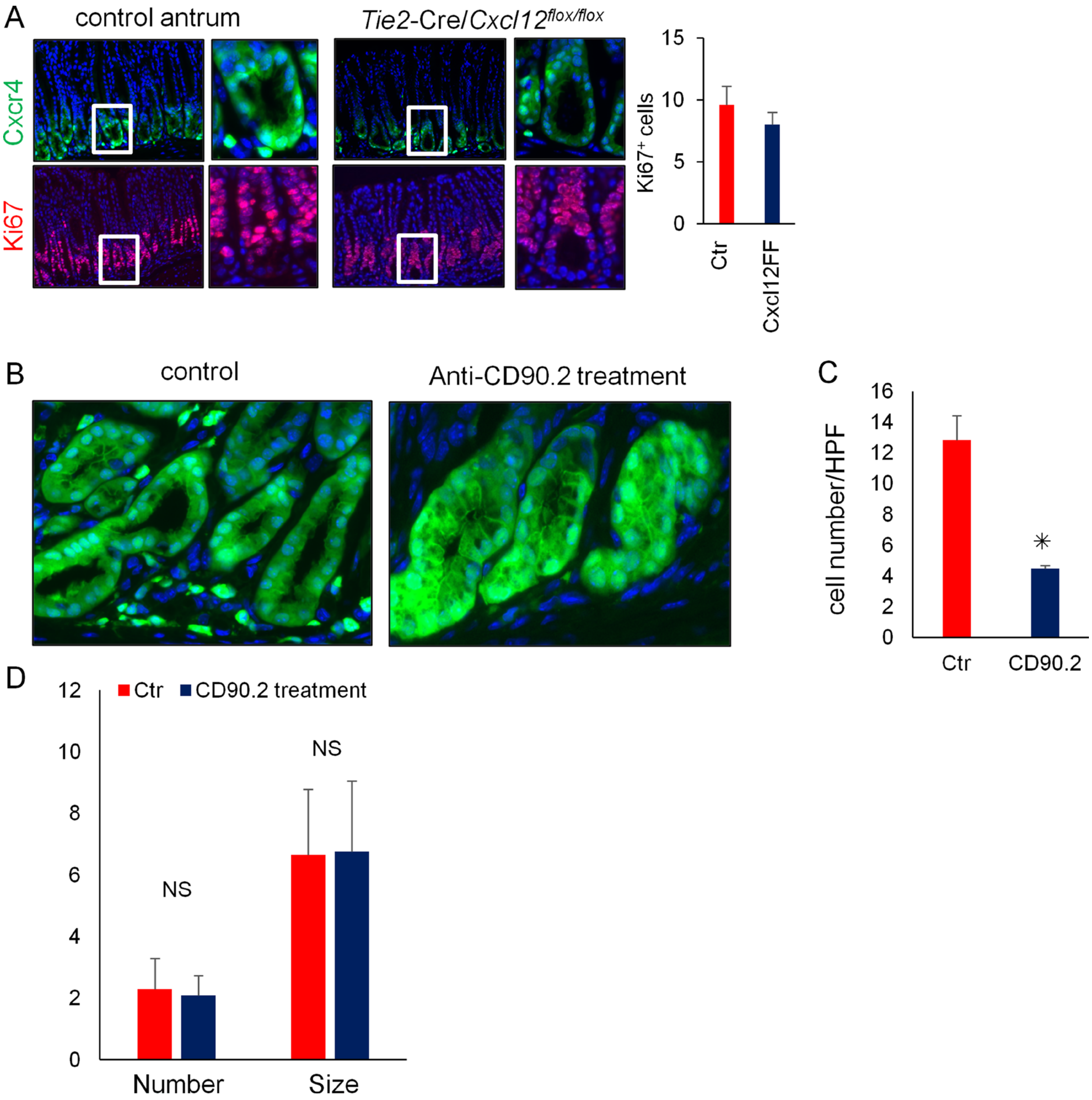

Supplementary Figure 5: The effects of Cxcr4 signaling in antral carcinogenesis. (**A**) Immunofluorescence of GFP (green) and Ki67 (red) in *Cxcr4*-EGFP; *Cxcl12*$^{flox/flox}$ (control) mice and *Tie2*-Cre; *Cxcr4*-EGFP; *Cxcl12*$^{flox/flox}$ mice. (Right) Ki67^{+} cells/gland were quantified. A total of 150 glands from three mice was analyzed. (**B**) MNU-induced antral tumor images of *Cxcr4*-EGFP mice without (control) and with anti-CD90.2 antibody treatment. (**C**) Cxcr4^{+} cells/gland in stroma of the MNU-derived antral tumor of *Cxcr4*-EGFP mice without (control) and with anti-CD90.2 antibody treatment. The total 150 glands from 3 mice/group are analyzed. (**D**) Macroscopic antral tumor numbers and sizes of Cxcr4-EGFP mice without (control, $N = 10$) and with 2 weeks anti-CD90.2 antibody treatment ($N = 8$) 40 weeks after the start of 5 cycles of MNU treatment.
